# The role of remote ischaemic preconditioning (RIPC) in colorectal surgery: a meta-analysis of randomized-controlled studies

**DOI:** 10.1007/s00423-025-03864-9

**Published:** 2025-09-08

**Authors:** Maria Chara Stylianidi, Sascha Vaghiri, Peter C. Ambe, Wolfram Trudo Knoefel, Dimitrios Prassas

**Affiliations:** 1 Department of Surgery (A), Medical Faculty, Heinrich-Heine-University, University Hospital Duesseldorf, Duesseldorf, Germany; 2 Department of Surgery II, Witten/Herdecke University, Witten, Germany; 3 Department of Surgery, , Katholisches Klinikum Essen, Teaching Hospital of Duisburg-Essen University, Philippusstift, Essen, Germany; 4 Department of Surgery (A), Medical Faculty, Heinrich- Heine-University, University Hospital Duesseldorf, Moorenstr. 5, Bldg. 12.46, Duesseldorf, 40225 Germany; 5 Department of Surgery, Katholisches Klinikum Essen, Teaching Hospital of Duisburg-Essen University, Huelsmannstrasse 17, Philippusstift, Essen, 45355 Germany

**Keywords:** Postoperative ileus, RIPC, Colorectal surgery, Morbidity

## Abstract

**Introduction:**

Remote ischaemic preconditioning (RIPC) which consists of repeated brief episodes of non-lethal limb ischaemia is associated with organ protection and improved clinical outcomes through complex pathophysiological pathways. The aim of this meta-analysis was to evaluate the postoperative effects of RIPC in bowel recovery and surgical morbidity after colorectal surgery.

**Methods:**

In strict adherence to the PRISMA guidelines, a systematic literature search was performed for studies comparing the postoperative effect RIPC in colorectal surgery. Data from eligible studies were extracted, qualitatively assessed, and included. Odds ratios (OR) and standardized mean differences (SMDs) with 95% confidence intervals (CIs) were calculated.

**Results:**

Four studies with a total of 311 patients were included. RIPC resulted in reduced rates of postoperative ileus (POI) (OR 0.42, 95% CI 0.21–0.85, *p* = 0.02) and lower postoperative TNF-α levels (SMD − 1.01, 95% CI -1.59,-0.43, *p* = 0.0007). There were no significant differences between the two groups in other clinical outcomes such as anastomotic leak, surgical morbidity and length of hospital stay.

**Conclusions:**

RIPC demonstrated significantly reduced POI rates and TNF-α levels in colorectal surgery and could be a potential supportive strategy to promote less tissue trauma and thus enhance bowel recovery. Larger randomized controlled trials with standardized study protocols are needed to validate the results presented here.

**Supplementary Information:**

The online version contains supplementary material available at 10.1007/s00423-025-03864-9.

## Introduction

Remote ischaemic preconditioning (RIPC), first presented in 1993 by Przyklenk et al. in an ischaemic dog heart model, is a phenomenon in which repeated, non-lethal episodes of ischaemia to an organ or limb can protect against subsequent ischaemia-reperfusion (I/R) injury in distant organs [1]. Many studies in the literature have shown the protective effect of RIPC in a target organ, such as the brain, myocardium, liver, intestine and lungs as a result of the reduction of inflammation and oxidative stress measured by anti-inflammatory agents such as TNF- α, interleukins, cytokines, HMGB1 and others [2–6]. The underlying mechanisms of RIPC are not fully understood, but based on current knowledge, information transfer involves neural, humoral and systemic pathways [7]. In addition, the positive cardioprotective effect of RIPC in cardiac surgery has been demonstrated, resulting in reduced release of myocardial injury biomarkers and thus an improved prognosis for patients [8–10]. However, its routine application in the daily context is limited as several consecutive studies failed to prove a consistent benefit of RIPC in cardioprotection and clinical outcomes [11–13]. At the same time, studies analysing vascular and non-vascular abdominal procedures such as hepatic resection show inconclusive results [14–18]. Furthermore, in experimental animal studies, RIPC did not significantly affect enhanced anastomotic bowel healing [19, 20]. Postoperative ileus (POI) is a common iatrogenic condition following abdominal surgery that slows patient recovery and increases the length and cost of hospitalization after surgery [21, 22]. Despite the introduction of enhanced recovery protocols such as ERAS, the incidence of postoperative ileus still lies between 10 and 30% [23, 24]. The pathophysiology of POI is not fully understood because of its multifactorial nature, including neurohormonal, inflammatory and pharmacological factors [25]. Another important complication in colorectal surgery with broad range impact is anastomotic leakage (AL), a defect in the bowel wall at the site of the anastomosis that leads to communication between the extra- and intraluminal spaces [26]. The incidence of anastomotic leak varies from 1 to 19% and has several risk factors such as type of anastomosis, local blood flow, ASA ≥ III, obesity, male gender, perianastomotic drain placement, prolonged operative time, emergency surgery, malnutrition, immunosuppression, and diabetes [27, 28].

At present, there is a relevant lack of pooled evidence that would justify the routine use of RIPC in colorectal surgery. Therefore, the aim of this meta-analysis was to evaluate the effect of RIPC in the outcomes of colorectal surgery and more specifically in the incidence of POI, AL, and overall morbidity.

## Materials and methods

This meta-analysis was performed according to the current PRISMA (Preferred Reporting Items for Systematic Reviews and Meta-Analyses) statement and according to the latest version of the Cochrane Handbook for Systematic Reviews of Interventions [29, 30].

Eligibility Criteria and Group Definition.

This meta-analysis includes all studies that compared the postoperative clinical outcomes of patients who underwent colorectal surgery after RIPC versus the control group who received Sham RIPC (comparator). The protocol of RIPC in the included was trials adapted based on previous animal and human proof of concept studies [1, 9]. To avoid heterogeneity, studies were selected for final analysis if they included patients with elective colorectal surgery for any reason. Outcomes of particular interest were overall postoperative morbidity and colorectal surgery specific complications including POI, AL, and length of postoperative hospital stay. Other analysed continuous parameters include GI-motility recovery indices such as, time to first stool or flatus, time to first solid diet and NG-tube reinsertion. Studies had to report at least one of the outcomes listed above to be included in the analysis. All types of published studies involving human participants within the defined inclusion criteria were considered eligible (e.g. randomized controlled trials (RCTs) and prospective or retrospective comparative cohort studies). Disagreements or differing conclusions in the selection of studies were resolved either by consensus or by consultation with an independent third author (D.P.).

### Literature search

Two authors (S.V. and D.P.) independently conducted the literature research that systematically collected all relevant studies up to September 2024 in the Pubmed (Medline), the Cochrane Central trials register, and google scholar databases. No langue or time restrictions were imposed. The following search terms were used in combination with the Boolean operators AND or OR: “remote ischemic preconditioning” AND (“colon” OR “rectum” OR “colorectal”). In addition, the reference list of retrieved articles (including systematic reviews, case reports, editorials, or experimental studies, which were excluded from the outset) was manually reviewed to identify potentially relevant citations for analysis. In case of duplicate or overlapping articles published by the same institution and authors, the most recent study was selected for inclusion.

### Data extraction and outcome measures

Two authors (S.V. and M.C.S.) independently abstracted all available and relevant data from studies meeting the inclusion criteria using a self-administered electronic data extraction form. Study, patient, and operative-specific information included country of origin, year of publication, study design, inclusion and exclusion criteria, enrolment period, RICP and control group protocols, number of patients enrolled per group and their demographics (age, sex, body mass index (BMI), (American society of anaesthesiologist) ASA class and comorbidities), indication for surgery, type of procedure, proportion of minimal invasive and open surgery cases, duration of surgery and anaesthesia (min), intraoperative blood loss, fluid administration, and urine output (ml). The primary endpoint was the rate of postoperative overall complications and AL. The secondary outcome analysis included the following objectives: GI-recovery parameters (time to first postoperative bowel movement, flatus, diet intake in hours), length of postoperative hospital stays (days), TNF-α levels at the first postoperative day (ng/ml), amount of intraoperative blood loss (ml), NG-tube reinsertion, reoperation, total parenteral nutrition, and postoperative ileus/GI-dysfunction rates. It should be noted that we have based our definitions of POI and AL on the respective studies and their definitions of these results (Table suppl. 1). Again, discrepancies in data extraction were resolved by consensus or reassessment by an independent third author (D.P.) to ensure consistency and accuracy.

### Quality and certainty assessment

The risk of bias of the for the included randomized trials was assessed independently by two authors (S.V. and D.P.) using the RoB 2 criteria [31]. Briefly, this recommended tool categorizes randomized trials into low to high risk of bias based on signalling questions derived from five potential bias domains (randomization process, deviations from the intended intervention, missing outcome data, measurement of the outcome, and selection of the reported results). The revised AMSTAR 2 instrument was used to critically appraise this meta-analysis [32]. The reviewers were not blinded to the study authors. Disagreements in the study bias assessment were discussed and resolved by consensus or consultation of a third author (M.C.S.). The Grading of Recommendations, Assessment, Development, and Evaluation (GRADE) methodology was applied to adequately document the strength and certainty of evidence using four levels for significant outcome parameters (high, moderate, low, and very low) [33].

### Statistical analyses

Statistical analysis was performed using RevMan software (version 5.3; Copenhagen: The Nordic Cochrane Centre, The Cochrane Collaboration, 2014). Pairwise meta-analyses were performed. For each endpoint of interest, summary treatment effect estimates with 95% confidence intervals (CIs) were calculated. For dichotomous endpoints, the odds ratio (OR) was chosen as the effect measure while standardized mean differences (SMDs) were calculated for continuous parameters. For continuous variables, the available data on medians and IQRs have been converted into means and standard deviations using the method proposed by Luo et al. [34]. Of note, continuous values were expressed in hours (time to first bowel movement/flatus/diet intake) or in days (length of hospital stay). Using the Cochrane Q test (chi-squared test; chi2) and the measurement of inconsistency (I^2^), the degree of heterogeneity among the included studies was interpreted as follows [29]: 0%−40% low Heterogeneity and may not be important, 30%−60% moderate Heterogeneity, 50%−90% substantial heterogeneity, >75% high heterogeneity [35]. When heterogeneity was low or moderate (I^2^ < 50%), summary estimates were calculated using a fixed-effects method. If I^2^ > 50%, the randomised model was used. If heterogeneity was low or moderate (I^2^ < 50%), summary estimates were calculated using a fixed-effects method. Where appropriate, subgroup analyses were performed to examine heterogeneity in the results. Tests for publication bias or funnel plots were omitted because of the small number of included studies as recommended. P-values < 0.05 of pooled data were considered significant.

## Results

### Study and patient characteristics

**Fig. 1:**
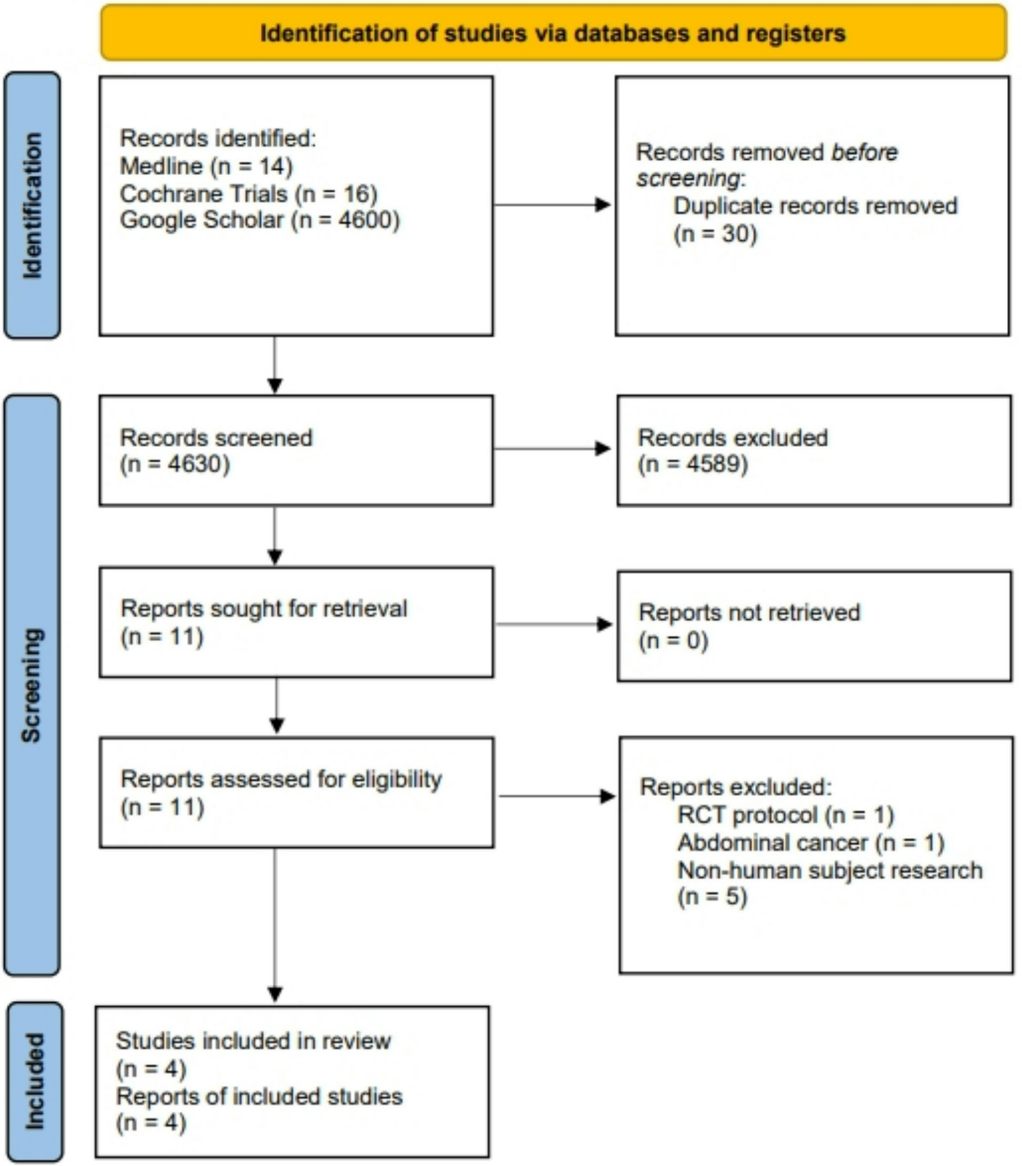
PRISMA diagram of study identification and selection for review analysis

The initial database query Yielded in 4630 results. After critical review and selection of the included reports, 11 full-text articles were screened for eligibility and four randomized monocentric-studies were included in the final qualitative and quantitative data analysis [36–39]. The detailed selection process is depicted in the PRISMA Flowchart (Fig. 1).

A total of 311 patients (RIPC: *n* = 156, Control: *n* = 155) form the final study cohort. Three studies originated from China [36–38], while one study was from Germany [39]. In all studies, the intervention of interest (RIPC) was performed based on the same protocol of three cycles of five min upper arm cuff inflation (pressure 200 mmHg) followed by a five min deflation period before skin incision. In the control group, the cuff was left deflated. All the study participants including the medical stuff and study assessors were blinded. Adherence to fast track postoperative recovery protocols was documented in two studies [36, 39]. All patients underwent elective colorectal cancer surgery. Two studies only included minimally invasive cases [36, 37], one study just an open approach [38], and one study a mixture of open and minimally invasive cases [39]. Left-sided and rectal resections were performed most frequently (73.3%) followed by right-sided colectomies (23.5%) and total/subtotal colectomies (3.2%). In one study the side of resection was not available [38]. The study, patient-and operative characteristics are summarized in detail in Tables 1 and 2.

**Table 1:** Study characteristics and protocols

Author	Year	Origin	Study period	Study design	Trial Registry	Patients included	Sample size analyzed	Masking	Exclusion Criteria	Fast-Track Protocol	Intervention RIPC	Intervention Start	Comparator	Primary Outcomes
Yang et al. (36)	2023	China	Dec 2020-Jun 2022	prospective-randomized	ChiCTR2100043313	80	77	Yes	ASA ≥ IV, active IBD, preoperative IVN, significant renal-hepatic impairment, planned enterostomy, gut dysmotility, language barrier, delirium/cognitive impairment, vascular disease	ERAS	Total 30 min: Upper limb/200 mmHg (3 × 5 min ischaemia)	Before surgery	Sham RIPC (deflated cuff)	Time for gastrointestinal tolerance, PPOI
Yi et al. (37)	2023	China	Mar 2022- Oct 2022	prospective-randomized	ChiCTR2200057892	100	87	Yes	Unable to communicate, relevant respiratory, circulatory, hepatic, renal, and endocrine diseases, peripheral vascular disease, neurological disorders, coagulation abnormality/low platelet count, upper limb thrombosis	No	Total 30 min: Upper limb/200 mmHg (3 × 5 min ischaemia)	Before surgery	Sham RIPC (simulated cuff inflation)	Gastrointestinal function I-FEED score on POD 3
He et al. (38)	2017	China	Oct 2015-Jun 2016	randomized, placebo-controlled	ChiCTR-IPR-15,007,287	90	90	Yes	MoCA score < 26, schizophrenia, dementia, epilepsy, parkinson, Alzheimer disease, brain surgery, serious hepatic or renal dysfunction, cardiac dysfunction, diabetes mellitus, right upper limb abnormalities, systolic pressure > 170 mmHg before RIPC, 18.5 < BMI > 28 kg/m^2^, non-chinese speaker	NA	Total 30 min: Upper limb/200 mmHg (3 × 5 min ischaemia)	Before surgery	Sham RIPC (deflated cuff)	MoCA score on POD 1
Hardt et al. (39)	2024	Germany	Oct 2019 – Jun 2022	pilot randomized controlled, triple-blind, monocenter trial	DRKS00018942	54	54	Yes	peripheral arterial disease, infections or wounds on the upper extremity, poorly controlled diabetes mellitus or upper limb deep vein thrombosis, inability to perform informed consent	ERAS	Total 30 min: Upper limb/200 mmHg (3 × 5 min ischaemia)	Before surgery	Sham RIPC (deflated cuff)	Anastomotic leak

*RIPC* Remote ischemic preconditioning, *ASA* American Society of Anesthesiologists, *BMI* Body Mass Index, *IBD* Inflammatory bowel disease, *IVN* Intravenous nutrition, *ERAS* Enhanced Recovery after Surgery, *PCIA* patient-controlled intravenous analgesia, *TAP* transversus abdomins plane block, *MoCA* Montral cognitive assessment, *NA* not available, *POD* Postoperative day, *PPOI *Prolonged postoperative ileus

**Table 2:** Patient and operative characteristics

Author	Groups	No. of patients	Age (years) mean ± SD	Gender (M/F)	BMI (kg/m^2^) mean ± SD	ASA score	Type of Procedure	MIS/ Open	Duration of Surgery (min) mean ± SD	Duration of Anaesthesia (min) mean ± SD
Yang et al. (36)	RIPC	40	55.42 ± 1.32	27/13	23.8 ± 3.4	I: 6 II: 23 III: 11	Right-sided: 18 Left-sided/rectal: 19 Total colectomy: 3	40/0	206.83 ± 19.40	NA
	Control	40	55.17 ± 2.75	24/16	23.8 ± 3.4	I: 5 II: 25 III: 10	Right-sided: 15 Left-sided/rectal: 21 Total colectomy:4	40/0	206.89 ± 33.87	NA
Yi et al. (37)	RIPC	44	64.6 ± 6.8	23/21	23.1 ± 2.6	I: 0 II: 33 III: 11	Right-sided: 9 Left-sided/rectal: 35	44/0	167.8 ± 61.1	199.9 ± 59.4
	Control	43	63.9 ± 7.7	24/19	24.0 ± 2.8	I: 1 II:32 III:10	Right-sided: 10 Left-sided/rectal: 33	43/0	165.5 ± 46.1	203.9 ± 49.1
He et al. (38)	RIPC	45	68.73 ± 2.89	23/22	23.63 ± 1.43	II: 12 III: 33	NA	0/45	102.56 ± 8.83	136.04 ± 7.65
	Control	45	68.33 ± 3.21	28/17	23.71 ± 0.99	II: 16 III: 29	NA	0/45	105.67 ± 10.15	138.51 ± 8.77
Hardt et al. (39)	RIPC	27	58.85 ± 11.8	22/5	26.135 ± 6.030	I: 4 II:19 III:4	Right-sided: 0 Left-sided/rectal*: 27	22/5	374.11 ± 131.55	NA
	Control	27	63.7 ± 13.32	15/12	27.834 ± 6.87	I: 3 II:19 III:5	Right-sided: 0 Left-sided/rectal*: 27	25/2	63.3 ± 133.06	NA

*RIPC* Remote ischemic preconditioning, *ASA* American Society of Anesthesiologists, *BMI* Body Mass Index, *NA* Not available, *MIS* minimally invasive * only (low) anterior and abdominoperineal resection

### Study quality and risk of bias

**Fig. 2:**
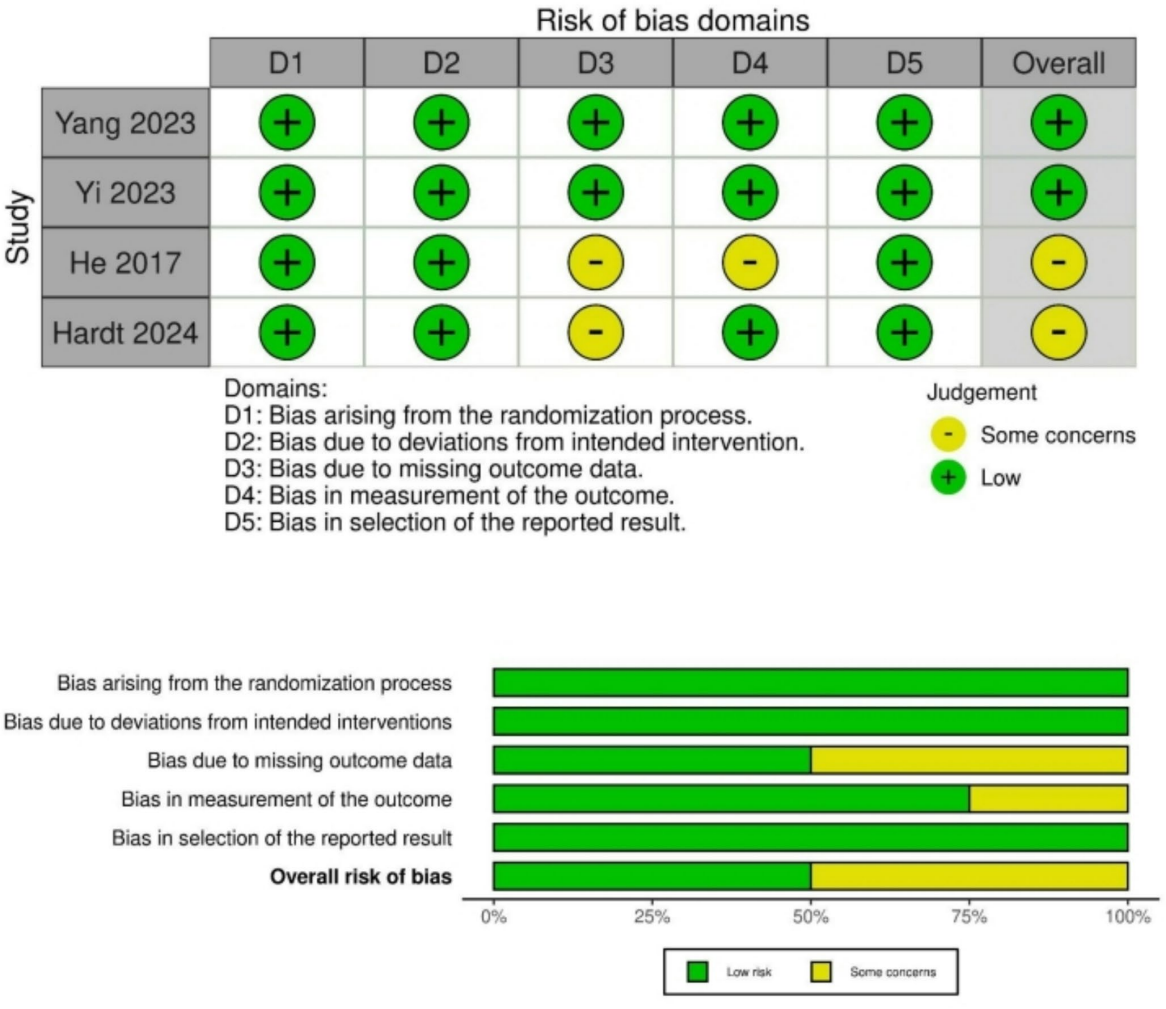
Risk of bias summary of the included studies based on the RoB 2 tool

The overall risk of bias in the four included randomized studies was low with some concerns in in studies by He et al. and Hardt et al. (Fig. 2) [38, 39]. The methodological quality of the present meta-analysis was determined as `high` using the AMSTAR 2 quality assessment tool. Of note, the definition of GI-recovery and POI outcomes varied among the included studies, and not all outcomes of interest were available in each study.

### Outcome analysis

#### Primary endpoints

**Fig. 3:**
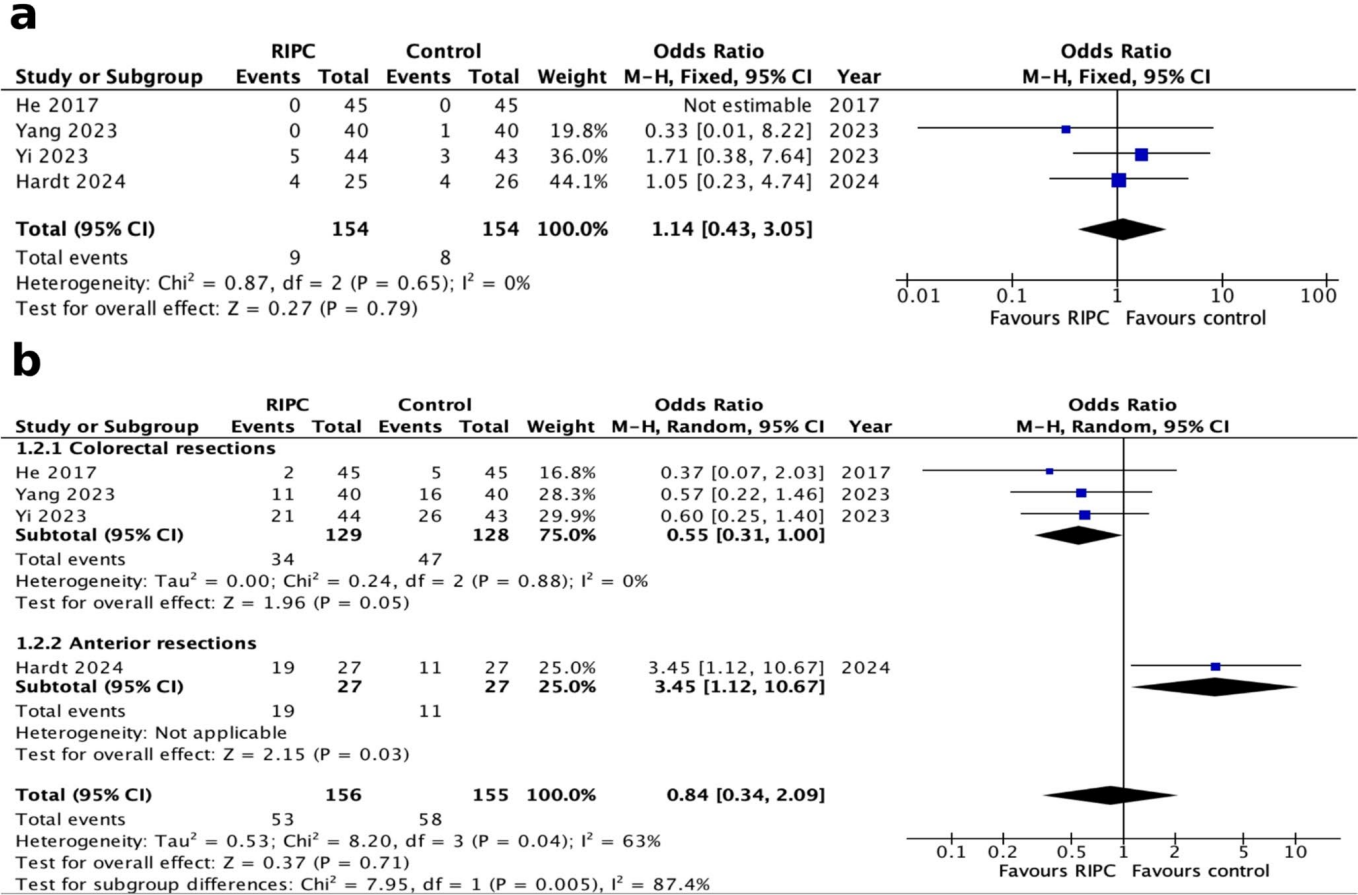
Forest plots of primary outcomes (RIPC versus control): (**a**) anastomotic leak and (**b**) overall morbidity

All studies reported the outcomes of primary interest with a total of 311 patients [36–39]. Meta-analysis demonstrated no statistically significant difference between the two groups regarding AL (OR 1.14, 95% CI 0.43–3.05, *p* = 0.79). The level of heterogeneity was notably low (I^2^ = 0%, Chi2 test: *p* = 0.65). Furthermore, pooled meta-analysis revealed no statistically significant superiority of RIPC versus control in terms of overall morbidity (OR 0.84, 95% CI 0.34–2.09, *p* = 0.71). Of note, the level of heterogeneity was substantial (I^2^ = 63%, Chi2 test: *p* = 0.04). Subsequent subgroup analysis demonstrated that studies with exclusively anterior rectal and abdominoperineal resections [39] do not appear to benefit from RIPC (OR 0.55, 95% CI 0.31–1.00, *p* = 0.05). The heterogeneity level was low (I^2^ = 0%, Chi2 test: *p* = 0.88) (Fig. 3a and b).

#### Secondary outcomes

**Fig. 4:**
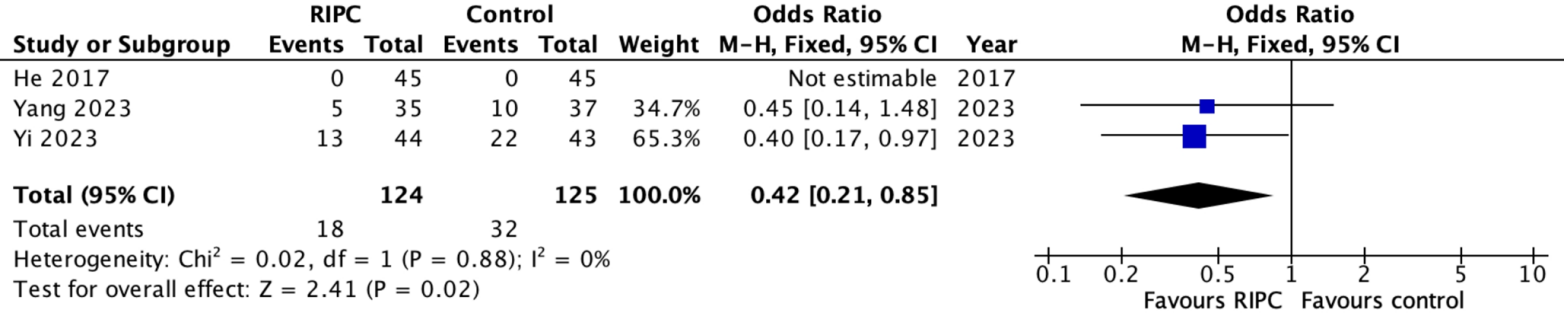
Forest plot of secondary outcome (RIPC versus control): Postoperative ileus

The results of the secondary outcomes analysis revealed a significantly lower rate of POI in the RIPC group with 249 patients from three studies (OR 0.42, 95% CI 0.21–0.85, *p* = 0.02) (Fig. 4) [36–38]. The level of heterogeneity was low (I^2^ = 0%, Chi2 test: *p* = 0.88) and the certainty of evidence according to GRADE was moderate (Table suppl. 2).

**Fig. 5:**
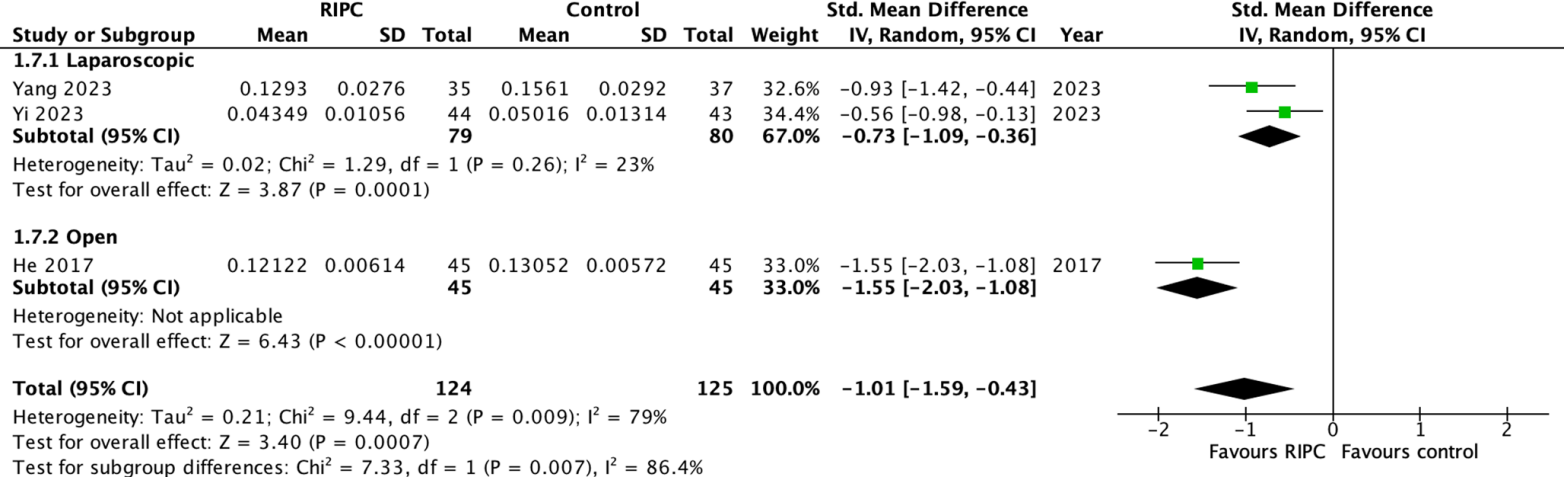
Forest plot of secondary outcome (RIPC versus control): TNF-α level on POD1

TNF-α levels on postoperative day one were reported in three studies with 149 patients [36–38]. A statistically significant difference was noted favouring the patient group with RIPC (SMD = −1.01; 95% CI −1.59 to −0.43, *p* = 0.0007; I^2^ = 79%, Chi2 test: *p* = 0.009). The source of heterogeneity was identified in the study by He et al. with open cases [38]. The subsequent subgroup with low heterogeneity still demonstrated a statistically significant effect favoring the RIPC patient group (SMD = −0.73; 95% CI −1.09 to −0.36, *p* = 0.0001; I^2^ = 23%, Chi2 test: *p* = 0.26) (Fig. 5). Noteworthy, based on GRADE judgement the level of evidence was low (Table suppl. 2). Analysis of other secondary endpoints including GI-recovery parameters (time to first bowel movement/flatus/diet intake), intraoperative blood loss, length of hospital stay, reoperation, NG-tube reinsertion, and total parenteral nutrition demonstrated no statistically significant difference between the RIPC and control groups. A detailed summary is shown in Table 3.

**Table 3:** Non-significant secondary outcomes

Outcomes	No. of included studies	No. of included patients		SMD/OR [95% CI]	*P*-value	Heterogeneity Level	
		RIPC	Control			I² (%)	*P*-value
Length of hospital stay (days)	3 (36,38,39)	107	109	−0.19 [−0.46, 0.08]	0.16	0	0.76
NG Tube reinsertion	3 (36,38,39)	112	112	0.77 [0.28, 2.10]	0.61	0	0.36
Reoperation	4 (36,37,38,39)	156	155	1.00 [0.14, 7.32]	1.00	0	1.00
Time to first bowel movement (hours)	2 (36,37)	79	80	−0.70 [−2.12, 0.73]	0.34	94	< 0.0001
Time to first flatus (hours)	2 (36,37)	84	83	0.19 [−0.51, 0.90]	0.59	81	0.02
Time to first solid diet (hours)	2 (36,37)	84	83	−0.51 [−1.63, 0.60]	0.37	92	0.0004
Total parenteral nutrition	2 (36,38)	85	85	1.37 [0.29, 6.56]	0.69	NA	NA
Blood loss intraoperatively (ml)	4 (36,37,38,39)	156	155	−0.04 [−0.46, 0.38]	0.85	71	0.02

*OR* Odds ratio, *SMD* Standardized mean difference, *NA* not applicable, *NG-tube* nasogastric tube

## Discussion

To the best of our knowledge, this is the first meta-analysis to investigate the role of RIPC on outcomes in colorectal surgery, with a particular focus on postoperative ileus and anastomotic leakage. The results of this meta-analysis with four included studies indicate that there was a significantly lower rate of POI in the RIPC group and no difference between the control and the RIPC groups in terms of AL and overall morbidity. Furthermore, our subgroup analysis revealed that the potential effect of RIPC on lowering postoperative morbidities diminished if just rectal resections were performed. Interestingly, despite the statistically significant results for POI, there was no difference between the control and RIPC groups in other GI-recovery parameters such as time to first bowel movement/flatus/diet intake. In addition, no significant effect on clinical outcomes including length of hospital stay, intraoperative blood loss, reoperation, NG tube reinsertion and total parenteral nutrition was observed in patients with RIPC. Regarding TNF-α levels, our analysis showed favourable results for the RIPC group by means of reduced postoperative inflammatory cytokine release.

In surgical procedures, RIPC has been studied primarily in cardiovascular surgery. A benchmark study published by Hausenloy et al. demonstrated that adult patients undergoing elective coronary artery bypass graft surgery could benefit from remote ischaemic preconditioning, as RIPC resulted in lower troponin levels [9]. Xie et al. showed in a meta-analysis of 30 included studies that RIPC reduces troponin I/troponin T release after cardiac surgery [40]. Stather et al. examined the effect of RIPC in vascular surgery. In their meta-analysis of 13 studies, they found that RIPC did not significantly affect mortality, renal dysfunction, myocardial infarction, myocardial injury or length of stay [41]. One randomized control trial by Papadopoulou et al. demonstrated a positive effect of RIPC on postoperative surgical and pulmonary complications but no impact in cardiac morbidity in patients with intra-abdominal cancer surgery [14]. Antonowicz et al. conducted a double-blinded, sham-controlled trial in patients undergoing elective gastrointestinal surgery or complex abdominal wall surgery to investigate the effect of RIPC on perioperative myocardial injury. In this study, RIPC did not reduce the incidence or severity of perioperative myocardial injury [42]. Van Zeggeren et al. showed in a randomised clinical trial that there is no effect of RIPC in postoperative cardiac and inflammatory biomarkers in patients undergoing pancreatic surgery [43]. A topic of interest in many recent published studies has been the effect of RIPC in liver surgery as local ischemic preconditioning has been shown to be a protective strategy against hepatic ischemia-reperfusion injury during hepatectomy. Zhang et al. showed in a meta-analysis of seven studies that alanine aminotransferase (ALT) and Aspartat-Aminotransferase (AST) levels in patients undergoing liver resection were lower in the RIPC group. There was no difference in bilirubin levels. However, as noted by the authors, the studies included in this meta-analysis were very heterogeneous and therefore these results should be interpreted with caution [44].

Currently, to our knowledge only the four studies included in this meta-analysis explicitly investigate the role of RIPC in colorectal surgery. The results on the effect of RIPC on anastomotic integrity are similar to the results of the published animal studies [19, 20]. Of note the effect of RIPC on POI and GI-dysfunction rates were reported in three studies [36–38], while time to first bowel movement and first flatus were mentioned in just two studies [36, 37]. Therefore, meta-analysis of the continuous GI-recovery parameters was not meaningful and conclusive. The prokinetic effect on the intestine is possibly due to the anti-inflammatory mechanisms induced by RIPC [37]. Indeed, it has been demonstrated that inflammatory markers in the abdominal exudate (such as procalcitonin (PCT), TNF- α, IL-6, and IL-1ß) were elevated in the scenario of POI after colorectal surgery [45, 46]. TNF-α level measurement was reported in three of the included studies [36–38]. Pooled meta-analysis showed that the expression of TNF-α at the first postoperative day was significantly lower in the RIPC group compared to the control group. This effect was even more pronounced and homogenous in the subgroup of patients undergoing minimally invasive approaches. Furthermore, these results are similar with a recently published meta-analysis indicating lower inflammatory reactions in laparoscopic surgery due to less severe operative trauma [47]. Of note, RIPC is a harmless procedure with very low reported side effects [48, 49]. Only in one vascular trial, RIPC was associated with acute ischaemic complications in invasive lower limb arterial occlusion [50]. Another important fact to mention is the extent to which anaesthetic regimens and confounders such as drugs and patients characteristics could attenuate the RIPC effect on organ protection [51–53]. The limited data provided in the four analyzed studies did not allow us to investigate these aspects.

When interpreting the results, several limitations must be taken into account: firstly, despite randomisation, the included studies have a small sample size (median sample size: RIPC *n* = 42, control *n* = 41.5). In addition, the exclusion criteria of the trials varied considerably within the monocentric design setting. Although the trials included only colorectal patients, the proportion of open versus minimally invasive procedures, the extent of surgical resection, and the type of anastomosis performed, which are important factors in the development of ileus, were not evenly distributed and not fully displayed respectively. In addition, the included trials showed considerable heterogeneity in reporting gastrointestinal motility parameters and two trials did not use an enhanced recovery protocol. For example, the I-FEED score was only applied in one study [37] to define postoperative GI-recovery while the proposed composite GI-2 outcome [54] as a validated and evidence-based measure was not used in any of the included studies.

## Conclusion

Although the effect of remote ischaemic preconditioning has been extensively studied in many cardiac and non-cardiac procedures, its value in colorectal surgery remains undetermined. However, RIPC demonstrated significantly reduced POI rates and TNF-α levels after colorectal surgery and could be a potential supportive strategy to promote less tissue trauma and thus enhanced bowel recovery. Larger-scaled high quality RCT’s are needed to ensure in depth exploration of the RIPC effect in colorectal surgery.

## Supplementary Information

Below is the link to the electronic supplementary material.

Supplementary Material 1 (DOCX 31.6 KB)

Supplementary Material 2 (DOCX 16.0 KB)

Supplementary Material 3 (DOXC 28.6 KB)

## Data Availability

No datasets were generated or analysed during the current study.
