# Supplementary material for: The role of remote ischaemic preconditioning (RIPC) in colorectal surgery: a meta-analysis of randomized-controlled studies
Source: Langenbecks Arch Surg. 2025 Sep 8;410(1):268. doi: 10.1007/s00423-025-03864-9 (PMC12420691; doi:10.1007/s00423-025-03864-9)
Supplement: Supplementary file 2 — Supplementary Material 2 (DOCX 16.0 KB) [file 423_2025_3864_MOESM2_ESM.docx]

| Table suppl. 1: Definition of AL and POI in the included studies | | |
| --- | --- | --- |
|  | POI | AL |
| Yang et al. (36) | ≥ 2 symptoms after POD 4:  - Inability to tolerate oral diet over the past 24 h  - Nausea or vomiting  - No flatus over the past 24 h  - Abdominal distension  - radiological evidence of intestinal distension without mechanical obstruction | Not specified |
| Yi et al. (37) | I-FEED score [1]:  - POGI (postoperative gastrointestinal intolerance:  3–5 points  - POGD (postoperative gastrointestinal dysfunction: ≥6 points | Not specified |
| He et al. (38) | Not specified | Not specified |
| Hardt et al. (39) | Not reported | Based on the definition of the International Study Group  of Rectal Cancer [2]:  Grade A, B, C  Confirmed by endoscopy or radiology |
| AL: anastomotic leakage, POI: postoperative ileus | | |

References:

1. Hedrick TL, McEvoy MD, Mythen MMG, et al (2018) American Society for Enhanced Recovery and Perioperative Quality Initiative Joint Consensus Statement on Postoperative Gastrointestinal Dysfunction Within an Enhanced Recovery Pathway for Elective Colorectal Surgery. Anesth Analg 126:1896–1907. https://doi.org/10.1213/ANE.0000000000002742

2. Rahbari NN, Weitz J, Hohenberger W, et al (2010) Definition and grading of anastomotic leakage following anterior resection of the rectum: a proposal by the International Study Group of Rectal Cancer. Surgery 147:339–351. https://doi.org/10.1016/j.surg.2009.10.012
