# Supplementary material for: The role of remote ischaemic preconditioning (RIPC) in colorectal surgery: a meta-analysis of randomized-controlled studies
Source: Langenbecks Arch Surg. 2025 Sep 8;410(1):268. doi: 10.1007/s00423-025-03864-9 (PMC12420691; doi:10.1007/s00423-025-03864-9)
Supplement: Supplementary file 3 — Supplementary Material 3 (DOXC 28.6 KB) [file 423_2025_3864_MOESM3_ESM.docx]

Table suppl. 2: The GRADE Certainty assessment for the significant outcomes

| Outcomes | No. of studies | **No. of included patients** | | OR/SMD [95 % CI] | **Quality assessment** | | | | | Quality |
| --- | --- | --- | --- | --- | --- | --- | --- | --- | --- | --- |
|  |  | RIPC | Control |  | Risk of bias^a^ | Inconsistency | Indirectness | Imprecision | Publication bias |  |
| POI | 3 [36,37,38] | 124 | 125 | 0.42 [0.21–0.85] | Not serious | Not serious | No indirectness | No imprecision | NA | Moderate |
| TNF-α level | 3 [36,37,38] | 124 | 125 | -1.01 [-1.59 to -0.43] | Not serious | Serious | No indirectness | No imprecision | NA | Low |

NA: not applicable, SMD: standardized mean difference, POI: postoperative ileus ^a^ Risk of bias assessed using the RoB 2 tool.
